# Lack of Zika Virus and Other Recognized Flaviviruses among the Mosquito Vectors during and Post the Hajj Mass Gathering

**DOI:** 10.3390/ijerph18126275

**Published:** 2021-06-10

**Authors:** Saber Yezli, Muhammad Yasir, Yara Yassin, Afnan Almazrua, Tagreed Al-Subhi, Norah Othman, Abdiasiis Omar, Abdelmohsin Abdoon, Yousif Elamin, Abuzaid Abuzaid, Turki Bafaraj, Hassen Alzahrani, Sameer Almahmoodi, Hussam Alzahrani, Kingsley Bieh, Badriah Alotaibi, Anas Khan, Mohammed Alzahrani, Esam I. Azhar

**Affiliations:** 1 The Global Centre for Mass Gatherings Medicine, Ministry of Health, Riyadh 12341, Saudi Arabia; yara@yassin.com (Y.Y.); almazrua.a@hotmail.com (A.A.); kinslezor@gmail.com (K.B.); bmalotaibi@moh.gov.sa (B.A.); anaskhan@ksu.edu.sa (A.K.); 2 Special Infectious Agents Unit, King Fahd Medical Research Center, King Abdulaziz University, P.O. Box 128442, Jeddah 21362, Saudi Arabia; yamuhammad@kau.edu.sa (M.Y.); tlalsobhe@kau.edu.sa (T.A.-S.); naothman@kau.edu.sa (N.O.); 3 Medical Laboratory Technology Department, Faculty of Applied Medical Sciences, King Abdulaziz University, P.O. Box 128442, Jeddah 21362, Saudi Arabia; 4 Infection Control and Hospital Epidemiology Department, King Faisal Specialist Hospital and Research Centre, Riyadh 11564, Saudi Arabia; 5 General Directorate of Vector-Borne & Zoonotic Diseases, Ministry of Health, Riyadh 12613, Saudi Arabia; omarai@moh.gov.sa (A.O.); abdoon80@hotmail.com (A.A.); ydelamin@moh.gov.sa (Y.E.); abuzaidabdalla@yahoo.com (A.A.); MoHAlzahrani@moh.gov.sa (M.A.); 6 Vector-Born and Zoonotic Diseases Department, Public Health Administration, Ministry of Health, Makkah 24321, Saudi Arabia; dr.turkibafaraj@me.com (T.B.); abojana2120@gmail.com (S.A.); 7 Department of Clinical Laboratory, King Khalid University Hospital, Riyadh 12372, Saudi Arabia; mr.hassenzi@gmail.com; 8 Vision Colleges, Faculty of Medicine, Alfarabi College, Riyadh 13226, Saudi Arabia; mmzahrani702@gmail.com; 9 Department of Emergency Medicine, College of Medicine, King Saud University, Riyadh 12372, Saudi Arabia

**Keywords:** vector-borne disease, Zika, flavivirus, mosquito, mass gathering, Saudi Arabia

## Abstract

Makkah city, Kingdom of Saudi Arabia (KSA), contains many of the world’s mosquito vectors of parasitic and arboviral disease and is the site of the Hajj mass gathering. As such there is a risk of exportation and globalization of vector-borne viruses, including the re-emerging Zika virus (ZIKV). There was international concern regarding the introduction of ZIKV to KSA and potential international spread of the virus following the 2016 Hajj which took place few days after the Rio summer Olympics at the height of the ZIKV pandemic. We aimed to detect flaviviruses, including ZIKV, circulating among mosquito hosts in the city of Makkah during and post the 2016 Hajj pilgrimage. Mosquitos (adults and larvae) were sampled from 15 sites in Makkah city during and post the 2016 Hajj and identified to species by morphological keys. Mosquitos were pooled according to date of collection, location, and species. A Pan-Flaviviruses RT-PCR assay that enables identification of 51 flaviviruses species and three tentative species was used to detect flavivirus RNA directly from mosquito homogenates. Between the 10 September and 6 October 2016, 9412 female mosquitos were collected. Of these, 81.3% were *Aedes aegypti*, 18.6% were *Culex* species, and 0.1% were *Anopheles* species. Of the total 493 mosquito pools generated, 242 (49%) were positive by the Pan-Flaviviruses primer set. Sequence analysis revealed that none of the mosquitos carried a pathogenic flavivirus, including ZIKV, but were infected with a novel insect-specific flavivirus. We found no pathogenic flaviviruses circulating in Makkah city during and post the 2016 Hajj and no evidence of introduction of ZIKV through the pilgrimage. Enhanced vector-borne diseases surveillance, prevention, and control are crucial in KSA especially during international mass gatherings such as the annual Hajj to prevent outbreaks and the spread of viruses with epidemic and pandemic potentials.

## 1. Introduction

*Flavivirus* is a genus of positive-sense RNA viruses with similar genomic organizations but fundamental differences in their natural host ranges and transmission cycles [1]. Most flaviviruses are horizontally transmitted between hematophagous arthropods (i.e., mosquitoes and ticks) and vertebrate hosts in which infections range from asymptomatic to severe or fatal hemorrhagic fever or neurological disease [2]. Important human pathogens include the mosquito-borne viruses; yellow fever virus (YFV), Zika virus (ZIKV), dengue virus (DENV), Japanese encephalitis virus (JEV), and West Nile virus (WNV)), and tick-borne encephalitis virus (TBEV). These pathogens have plagued mankind, accounting for millions of morbidity and mortality worldwide, and potentially could affect non-endemic areas already colonized by the vectors [3,4]. Factors such as population movements, unintentional transport of infected vectors, and travel to endemic areas have greatly contributed to the increase in flaviviral infections worldwide and their expansion to new territories [3].

The Kingdom of Saudi Arabia (KSA) is a host of many mosquito vectors for flaviviruses, especially in the South and Southwest regions of the country [5,6]. DENV is the most prevalent of the mosquito-borne viruses in KSA and numerous outbreaks of dengue fever have been reported in the Kingdom over the years [7]. Given that Saudi Arabia is also visited by millions of travelers each year, including up to 10 million religious pilgrims during the Hajj and Umrah mass gatherings, the risk of outbreaks and globalization of mosquito-borne flaviviruses, including DENV and ZIKV, is significant in KSA [8,9]. Since first discovered in 1947, ZIKV has caused major outbreaks in Micronesia, French Polynesia, and recently in Brazil, where it attracted global attention due to its quick spread to many other countries [10]. The bite of infected *Aedes aegypti* is the primary mode of transmission between humans, although transmission is also possible through other Aedes species and through non-vector borne routes [10,11]. While most ZIKV infections are asymptomatic or present mild clinical disease, ZIKV infection during pregnancy can cause congenital microcephaly and other brain defects; Guillain-Barre syndrome, stillbirth, and miscarriages [10]. Guillain-Barré syndrome, meningoencephalitis, and acute myelitis in adults have also been reported with ZIKV infection [11].

On 1 February 2016, the World Health Organization’s (WHO) International Health Regulations Emergency Committee declared the ZIKV outbreak a Public Health Emergency of International Concern [12]. There were calls by leading public health experts to postpone, cancel, or move the Rio summer’s Olympic Games in Brazil, scheduled for August 2016, for fear of accelerating a global spread of ZIKV [13]. The WHO backed the travel but advised athletes and visitors to take steps to manage health risks [13]. The WHO stating that canceling or relocating the Olympics would not significantly alter the international spread of ZIKV was supported by a growing body of literature indicating low personal effect of ZIKA on travelers [14]. However, there were still concerns regarding scheduled international mass gatherings in 2016 and their potential role in exacerbating the spread of ZIKV, especially given that ZIKV was thought to have been introduced to Brazil via an international sporting event in 2014 [15]. In particular, there was apprehension in relation to the Hajj mass gathering in KSA which took place few days after the Rio Olympics in September 2016 [9,16]. Hajj is attended by over 2 million pilgrims from up to 180 countries with around 7000 originating from Latin America [9]. Moreover, previous reports showed that larva of *Ae. aegypti* is present all year round near indoor habitats in cities like Makkah, Madinah, and Jeddah where pilgrims stay during Hajj and that the local environmental factors support all year risk for mosquitoes-borne diseases [5].

In this study, we aimed to collect mosquitoes throughout Makkah city during and after the 2016 Hajj and screen them for flaviviruses. The objectives were to determine the mosquito species and flaviviruses circulating in the city during the study period and investigate the possibility of the introduction of ZIKV to KSA via the 2016 Hajj mass gathering.

## 2. Methods

### 2.1. Study Area

**Figure 1:**
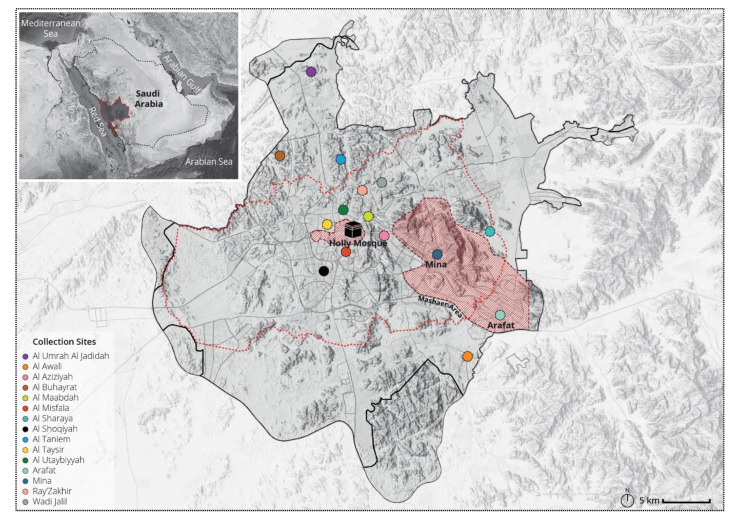
Location of Makkah Province and sites of mosquitoes and larva collection during and post the 2016 Hajj.

The Holy city of Makkah (21°25′21′′ N and 39°49′24′′ E) is located in the Makkah Province in the Western part of KSA (Figure 1). Makkah city lies at an elevation of 277 m above sea level, and approximately 80 Km inland from the Red Sea. The city is situated between mountains, which have defined the contemporary expansion of the city with a local population of over 1,800,000 in 2016. The city of Makkah centers on the Holy Mosque (Al-Haram), which is lower than most of the city. The area around the Holy Mosque comprises the old city, while other areas such as Mina and Arafat (Figure 1) are holy sites and part of the Hajj pilgrimage journey.

### 2.2. Mosquito Samples Collection

Mosquito sampling (adults and larvae) was conducted during and post the 2016 Hajj, between 10 September and 6 October 2016 corresponding to the 9 DulHijah-5 Muharram 1438H in the Islamic calendar. Adult mosquitoes were collected using CDC light traps, Black hole traps, and BioGents sentinel traps. Samples were collected from 15 sites within Makkah city (Figure 1). At each sampling site, 5–10 mosquito traps were set up at suitable intervals depending on the sites’ area and mosquito breeding locations. Mosquito traps were placed strategically close to animal markets and slaughterhouses, farms and human dwellings as conditions suitable for mosquito proliferation might be present. In addition, mosquito traps were also placed around the Holy Mosque.

Mosquito larvae were also collected from the same sampling sites. Due to a wide variety in type, size, and shapes of the water-holding containers, various equipment were used for sampling the immature stages of the container-breeding mosquitoes. If the container was large enough, a dipper or net was used. In the case of smaller containers, the entire contents were emptied onto trays and the immature stages picked out using a dropper.

Trapped mosquitoes and collected larvae were transported to the vector-borne diseases laboratory of the Saudi Ministry of Health in Makkah, where adult mosquitoes were exposed to freezing temperatures for 1 h to kill them. The immature stages were reared out to adults. Afterward, mosquitoes were sorted on chilled plates and identified to species by morphological keys. Mosquitoes of the same species, collection site, and collection date were pooled in groups of 15–20 mosquitoes, stored in dry ice, and then transported to the Special Infectious Agents Unit at King Fahad Research Center, King Abdulaziz University, Jeddah, for processing.

### 2.3. Nucleic Acid Extraction from Mosquito Samples

Total nucleic acid was extracted from the pools of mosquitoes using QIAamp Viral Nucleic Acid Purification Kits (Qiagen, Düsseldorf, Germany). Briefly, in each tube, the pool of mosquitoes to be tested was mixed together with 0.6 mL of sterilized RPMI culture medium, and homogenized using 1 bead of tungsten of 3-mm diameter (Qiagen, Düsseldorf, Germany) with TissueLyser (Qiagen, Düsseldorf, Germany). The tubes were then centrifuged at 1000 rpm to separate mosquitoes’ remains from the liquid supernatant. Virus Mini Kit (Qiagen, Düsseldorf, Germany) was used to extract the total nucleic acid (RNA and DNA). Necessary precautions were made to perform all the possible extraction steps at 4 °C. The extracted nucleic acid from each pool was stored at −80 °C until further processing.

### 2.4. Virus Detection and Identification in Mosquito Pools Homogenates

Extraction of RNA from the mosquito pools was confirmed by RT-PCR using actin-1 specific primers. The actin-1 gene was successfully amplified from the tested mosquitoes’ pools using primer set Act-2F (5′-ATGGTCGGYATGGGNCAGAAGGACTC-3′) and Act-8R (5′-GATTCCATACCCAGGAAG-GADGG-3′) following the protocol described previously [17]. The improved RT-PCR protocol developed by Moureau et al. [17] was used to detect Pan-Flaviviruses in the extracted nucleic acids from the collected mosquitoes’ pools. Briefly, one-step real-time RT-PCR was performed with SYBR Green using forward primer PF1S: 5-TGY-RTB-TA Y -AA C- ATG-ATG-GG and improved degenerate reverse primer PF2R-bis (5-GTG-TCC-CA I-CCNGCN-GTR- TC). Amplified PCR products were purified using Qiagen PCR purification and subjected to capillary sequencing (ABI PRISM 3700 DNA Analyzer, Applied Biosystems, Waltham, MA, USA) using the same amplification primers used for PCR. Sequence similarities of tested samples were performed using BLASTN algorithm with the NCBI GenBank database.

### 2.5. Confirmation That Mosquitoes Were Infected by Mosquito Flavivirus

To confirm that the mosquitoes in our pools were infected by a mosquito flavivirus, a TaqMan assay was designed to amplify specifically mosquito virus from the extracted nucleic acid pools. The primers (MqFlf-TGGAATATGAGGCCTTGGGCT), (MqFlr-AAATTTCCCGGGTGGCGTGG) and probe (CTCAACGAAGACCATTGGGTAGCC) were designed from the sequences that aligned with mosquito flavivirus in the blast analysis. The amplified product was then sequenced and compared to the products from the Pan-Flaviviruses assay. As the TaqMan assay product was small (72 bp) and could not be sequenced directly, it was first cloned into a PGMET Essay Vector (Promega, WI, USA). The ligated products were transformed into *E. coli* DH5α chemical competent strain using heat shock method following the manufacture guidelines (Promega, WI, USA). Transformation was confirmed in the *E. coli* strains by PCR using TaqMan assay primer set. Plasmid was extracted from the positive colonies and sequenced with T7 primer of PGMET vector using capillary sequencing (ABI PRISM 3700 DNA Analyzer, Applied Biosystems).

### 2.6. Institutional Review Board Statement

The study was approved by the King Fahad Medical City Ethics Committee and the Institutional Review Board (IRB log: 16-401E, approved on 6 September 2016).

## 3. Results

### 3.1. Mosquito Samples Collected

The study originally collected 8878 mosquitoes and 602 larvae. After rearing the larvae and mosquitoes’ identification and sorting, a final sample size of 9412 female mosquitoes was included in the study. Of these, 7650 (81.3%) were *Ae. aegypti*, 1749 (18.6%) were *Culex* species, and 13 (0.1%) were *Anopheles* species. These samples were used to generate 493 mosquito pools that underwent molecular processing.

### 3.2. Flaviviruses Detection in the Mosquito Pools Homogenates

Of the total 493 nucleic acid extractions, 242 (49%) were positive by Pan-Flaviviruses primer set. These comprised 221 (91.3%) pools of *Ae. aegypti* and 21 (8.7%) pools of *Culex* species.

### 3.3. Identification of Flaviviruses

**Figure 2:**
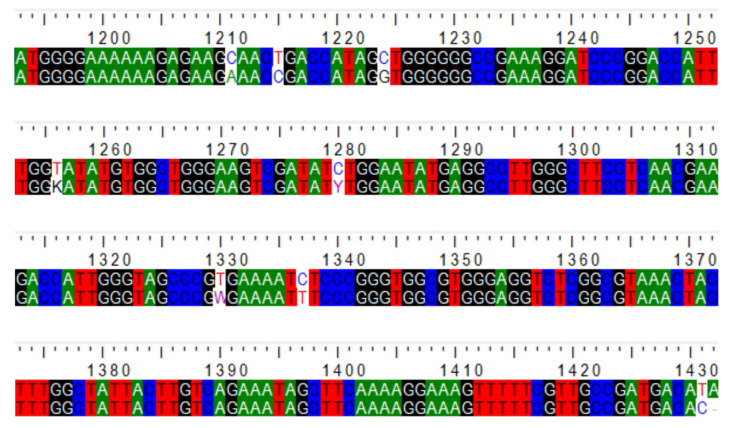
Alignment analysis of the query sequence (bottom) with mosquito-associated flavivirus (AY347953.2).

The RT-PCR protocol used is based on the amplification of a 269–272 region of the N terminal of the RNA-dependent RNA polymerase domain (NS5 gene) and enables the identification of 51 flavivirus species and 3 tentative species. However, the blast analysis of the amplified region revealed that the latter aligned with mosquito flavivirus strains and mosquito genome (Figure 2).

There were two possible explanations for this observation. Either the RNA-dependent RNA polymerase genome segment of flavivirus was integrated into the mosquito genome or the mosquitoes were infected with other mosquito viruses. In order to address these two issues, a PCR with Pan-Flaviviruses primer set was performed on the extracted nucleic acids (DNA) without cDNA synthesis. No amplification was observed, confirming that flavivirus genome was not integrated into the mosquitoes’ genome. These results also supported the hypothesis that mosquitoes in our study were infected with a novel mosquito-specific flavivirus.

### 3.4. Confirmation That Mosquitoes Were Infected by a Mosquito Flavivirus

To confirm that the mosquitoes in our pools were infected by a mosquito flavivirus, a TaqMan assay was designed to amplify specifically mosquito virus from the extracted nucleic acid pools. All the 242 Pan-Flavivirus-positive pool were positive by mosquito virus-specific TaqMan assay and had similar sequence. These results suggest that the collected pools of mosquitoes in our study were probably carrying a candidate novel mosquito-specific flavivirus.

## 4. Discussion

We conducted a screening of mosquito-borne flaviviruses among the vector in Makkah city, during and after the 2016 Hajj. Most of the collected mosquito vectors were *Ae. aegypti* with evidence of the presence of *Culex* and *Anopheles* species. Using our screening methodology, no known flaviviruses were detected including ZIKV. There was evidence that the mosquito vector was infected with a probably novel insect flavivirus. These results suggest that ZIKV was not circulating in Makkah during the study period and that there is no evidence that ZIKV was introduced to KSA through the 2016 Hajj mass gathering.

Mosquito-borne diseases are a significant health issue in KSA and several of these diseases have been documented in the country, including dengue fever, malaria, Rift Valley fever, and chikungunya [7,18,19,20]. In addition, WNV and Sindbis virus were isolated from mosquitoes in the country, demonstrating the potential for transmission [21,22]. The Saudi Ministry of Health reports (https://www.moh.gov.sa/en/Ministry/Statistics/book, accessed on 3 June 2021) indicate that with the exception of dengue fever, arboviral infections are uncommon in the country. For the last 10 years (2011–2020), there were no cases of yellow fever or Rift Valley fever in the Kingdom. In the same period, 406 cases of Khumra fever were reported in KSA with no clear seasonality and none of the cases occurred in Makkah. The flavivirus, DENV, is the most prevalent of the mosquito-borne viruses in KSA and has been documented in the country since the early 1990s [7,23]. Three serotypes of DENV (1, 2, and 3) are known to be endemic and circulating in KSA with DENV-2 being the predominant serotype [23]. A recent seroprevalence study using samples from Makkah in 2015–2016 also identified DENV-4 in the country [24]. Numerous dengue fever outbreaks have been reported in KSA over the years, and recent serological studies found seroprevalence rates of 37–47% [23]. According to the Saudi Ministry of Health (https://www.moh.gov.sa/en/Ministry/Statistics/book, accessed on 3 June 2021), few thousand cases of dengue fever are reported each year in the Kingdom with an incidence rate in the last 10 years ranging between 5.99 and 17.10 per 100,000 population. Most cases are reported in Jeddah, Makkah, and Jazan regions and occur between March and July.

Studies that investigated the mosquito fauna in KSA found a wide variety of mosquito species and great diversity in their breeding sites as well as a significant variation in the mosquito species composition by region [5,25,26]. Saudi Arabia contains many of the world’s mosquito vectors of parasitic and arboviral diseases [5,6], and the abundance of several mosquito species from the Afrotropical and Oriental regions increases the potential for mosquito-borne diseases present in those regions to spread into KSA. In the current study, we identified mosquito species belonging to three genera: *Culex, Aedes,* and *Anopheles,* in Makkah city. These mosquitoes can be vectors for a number of diseases, including malaria and arboviral diseases. *Ae. aegypti* was by far the most common species encountered. Previous studies from the Makkah region found similar results, albeit with different species composition to ours. For example, a survey conducted in Makkah between March 2004 and February 2006 caught 19 mosquito species belonging to *Culex, Aedes*, *Anopheles,* and *Culiseta* genera [27]. The abundance of these mosquito species was influenced by rainfall patterns and *Aedes* mosquitoes were most commonly found indoors. During a one-year (between April 2008 and March 2009) survey of households in Makkah city, Aziz et al. [28] collected 32,109 larvae from indoors and outdoors containers. These belonged to three genera: *Culex* and *Aedes* (most commonly encountered) and *Anopheles*. *Ae. aegypti* larval abundance exhibited marked temporal variations, overall, being usually more abundant during the wet season (May to October).

Despite most of the 9412 mosquitoes collected in this study being of the primary vector for ZIKV, the virus was not detected in any of the samples. Since first identified in the late 1940s, ZIKV has been documented in many regions of the world and caused numerous outbreaks worldwide [10,11]. Outbreaks of ZIKV infection peaked in 2016 and declined substantially through 2017 and 2018 in the America region. However, the spread of ZIKV and its potential re-emergence in countries with prior reports of virus transmission is a concern, given the ZIKV infection is associated with high frequency of neurologic complications and the current lack of available vaccine or therapeutic options [10]. By July 2019, 87 countries and territories across four of the six WHO regions have recorded autochthonous mosquito-borne transmission of ZIKV and it is likely that ZIKV will spread to more countries [10,29]. Saudi Arabia is among the countries with established ZIKV mosquito vectors but as of yet no known cases of ZIKV transmission in the Kingdom have been reported to the WHO [29]. Nevertheless, given the presence of the vector and documented imported cases of ZIKV around the globe [10], introduction and spread of ZIKV to KSA is a significant risk. This is particularly relevant given the yearly influx of travelers to the country, especially during the Hajj and Umrah mass gatherings, including pilgrims from ZIKA-endemic areas [8,9]. Our results support the notion that ZIKV was not introduced to KSA via the 2016 Hajj mass gathering and that the virus was not circulating among pilgrims and the local Makkah population during the study period.

Although half of our mosquito pools were positive by Pan-Flavivirus primers, none matched to any of the 51 flavivirus species and 3 tentative species in the assay [17]. These include the mosquito-borne viruses; DENV serotypes 1–4, YFV, WNV, and JEV. These results suggest mosquitoes were infected with a candidate novel insect-specific flavivirus. These results are not unique. A study from Spain collected 72,895 female mosquitoes between 2001 and 2005 and screened the resulting 4723 pools for arboviral RNA [30]. Similar to our results, no arboviral RNA from known pathogenic arboviruses was found. However, 111 pools tested positive for unknown mosquito flavivirus. New flaviviruses are constantly being described, and a number of novel insect-specific flaviviruses have been described in the recent years [31,32,33].

A number of factors could explain the results of the current study. The 2016 Hajj and the study period coincided with the dry season in Makkah, and this may have affected the number and type of mosquitoes collected and mosquito-borne viruses transmission. In the case of some viruses, including ZIKV and JEV, lack of detection may reflect the absence of the virus in the study location, as these have not yet been documented in the Kingdom. For viruses known to be endemic in the South and Southwest of KSA, such as DENV, the lack of detection among the mosquito samples may also be due to the comprehensive vector-borne diseases prevention programs implemented in the Kingdom and especially for the Hajj season. These include vector and disease surveillance, community-based educational and awareness campaigns, as well as vector control programme that includes routine widespread insecticides spraying. During Hajj, vector management and control is intensified in liaison with the Ministry of Health, Ministry of Agriculture, and Makkah municipality. This includes assessing pest control, identifying the zones and density of mosquitoes in the area, and undertaking insecticide spraying campaigns, including the aerial spraying of the holy sites of Mina and Arafat with long-lasting pesticide as well as many other locations in Makkah where pilgrims live or congregate [34]. In addition, the Saudi authorities require that pilgrims arriving from other countries or areas at risk of yellow fever transmission must present a valid yellow fever vaccination certificate [35]. Furthermore, The Saudi Ministry of Health recommends pilgrims take necessary measures to avoid mosquito bites during the day and evening, including wearing protective clothing, using physical barriers such as window screens and closed doors, and applying appropriate insect repellent to skin or clothing [35]. Furthermore, in accordance with the international health regulations, all aircraft and ship carriers are required to provide an official valid carrier sanitation certificate to verify that insecticide spraying was done before arrival at the port [34,35].

Our study has some limitations. We did not include positive controls for important known flaviviruses in our study. In addition, due to the expansion of unknown mosquito flaviviruses, the primers used in this study may have amplification uncertainty. As stated above, the study was conducted during the dry season, this may have affected the number, and type of mosquitoes collected given the adverse effect of high temperature and decreased rainfall on availability of oviposition sites, larval abundance, and lifespan. Aziz et al. [28] reported that the number of mosquito larvae in the wet season in Makkah was eight-fold that of the dry season, with a peak in March. Other factors could have impacted the number and species of mosquitoes collected in this study, including the routine governmental surveillance and insecticide spraying schemes in the region as well as the efficiency and positioning of the mosquito traps and species attractiveness to them.

## 5. Conclusions

In summary, we screened for flaviviruses among mosquitoes collected in Makkah during and after the 2016 Hajj at the height of the ZIKV epidemic. No known flaviviruses were detected, including ZIKV; however, mosquitoes were infected with a probably novel insect flavivirus. Given the international concern over the spread of ZIKV after the 2016 Brazil Olympics, our results do not show evidence that ZIKV was introduced to KSA via the Hajj, which took place a few days after the Olympics. International mass gatherings such as the Hajj and Umrah can facilitate the transmission of vector-borne diseases and the introduction and worldwide dissemination of the causative agents. With a significant expected increase in the number of travelers and religious visitors to KSA according to its 2030 vision, strategic planning and collaboration at multiple levels for vector-borne diseases prevention and control are required in the Kingdom. This includes expanded continuous surveillance and intensified vector management and control, increased reference laboratory diagnostic testing, and research and development for new diagnostics, therapeutics, and vaccines [8,36]. Further studies should investigate the mosquito virus identified in the current study, including isolation of the virus and whole-genome sequence analysis, which may reveal a new virus that is circulating in the local mosquitoes of Saudi Arabia.

## Data Availability

The dataset is available from the corresponding author on reasonable request.

## References

[1] Lindenbach B.D., Thiel H.J., Rice C.M., Knipe D.M., Howley P.M. (2007). Flaviviridae: The viruses and their replication. Fields Virology.

[2] Gubler D.J., Kuno G., Markoff L., Knipe D.M., Howley P.M. (2007). Flaviviruses. Fields Virology.

[3] Daep C.A., Munoz-Jordan J.L., Eugenin E.A. (2014). Flaviviruses, an expanding threat in public health: Focus on dengue, West Nile, and Japanese encephalitis virus. J. Neurovirol..

[4] Guarner J., Hale G.L. (2019). Four human diseases with significant public health impact caused by mosquito-borne flaviviruses: West Nile, Zika, dengue and yellow fever. Semin. Diagn. Pathol..

[5] Khater E.I., Sowilem M.M., Sallam M.F., Alahmed A.M. (2013). Ecology and habitat characterization of mosquitoes in Saudi Arabia. Trop. Med..

[6] Alahmed A.M., Munawar K., Khalil S.M.S., Harbach R.E. (2019). Assessment and an updated list of the mosquitoes of Saudi Arabia. Parasites Vectors.

[7] Alhaeli A., Bahkali S., Ali A., Househ M.S., El-Metwally A.A. (2016). The epidemiology of Dengue fever in Saudi Arabia: A systematic review. J. Infect. Public Health.

[8] Almutairi M.M., Alsalem W.S., Hassanain M., Hotez P.J. (2018). Hajj, Umrah, and the neglected tropical diseases. PLoS Negl. Trop. Dis..

[9] Elachola H., Gozzer E., Zhuo J., Memish Z.A. (2016). A crucial time for public health preparedness: Zika virus and the 2016 Olympics, Umrah, and Hajj. Lancet.

[10] Pielnaa P., Al-Saadawe M., Saro A., Dama M.F., Zhou M., Huang Y., Huang J., Xia Z. (2020). Zika virus-spread, epidemiology, genome, transmission cycle, clinical manifestation, associated challenges, vaccine and antiviral drug development. Virology.

[11] Masmejan S., Musso D., Vouga M., Pomar L., Dashraath P., Stojanov M., Panchaud A., Baud D. (2020). Zika Virus. Pathogens.

[12] World Health Organization In Proceedings of the WHO Statement on the First Meeting of the International Health Regulations (2005) (IHR 2005) Emergency Committee on Zika Virus and Observed Increase in Neurological Disorders and Neonatal Malformations, Teleconference.

[13] Coombes R. (2016). Call to cancel 2016 Olympics because of Zika risk is not backed by WHO guidance. BMJ.

[14] Lewnard J.A., Gonsalves G., Ko A.I. (2016). Low Risk of International Zika Virus Spread due to the 2016 Olympics in Brazil. Ann. Intern. Med..

[15] Petersen E., Wilson M.E., Touch S., McCloskey B., Mwaba P., Bates M., Dar O., Mattes F., Kidd M., Ippolito G. (2016). Rapid Spread of Zika Virus in The Americas--Implications for Public Health Preparedness for Mass Gatherings at the 2016 Brazil Olympic Games. Int. J. Infect. Dis..

[16] Ibrahim N.K. (2016). Zika virus: Epidemiology, current phobia and preparedness for upcoming mass gatherings, with examples from World Olympics and Pilgrimage. Pak. J. Med. Sci..

[17] Moureau G., Temmam S., Gonzalez J.P., Charrel R.N., Grard G., de Lamballerie X. (2007). A real-time RT-PCR method for the universal detection and identification of flaviviruses. Vector Borne Zoonotic Dis..

[18] Ahmad K. (2000). More deaths from Rift Valley fever in Saudi Arabia and Yemen. Lancet.

[19] Hussain R., Alomar I., Memish Z.A. (2013). Chikungunya virus: Emergence of an arthritic arbovirus in Jeddah, Saudi Arabia. East. Mediterr. Health J..

[20] Amer O.S., Waly M.I., Burhan I.W., Al-Malki E.S., Smida A., Al-Benasy K.S. (2020). Epidemiological trends of malaria in the Western regions of Saudi Arabia: A cross sectional study. J. Infect. Dev. Ctries..

[21] Wills W.M., Jakob W.L., Francy D.B., Oertley R.E., Anani E., Calisher C.H., Monath T.P. (1985). Sindbis virus isolations from Saudi Arabian mosquitoes. Trans. R. Soc. Trop. Med. Hyg..

[22] Al Ali K., Elbadry A.A., Eassa A.H., Aljuhan A.M., AlZubiany S.F., Ibrahim E.D. (2008). A Study on Culex Species and Culex Transmitted Diseases in AI-Madinah AI-Munawarah, Saudi Arabia. Parasitol. United J..

[23] Altassan K.K., Morin C., Shocket M.S., Ebi K., Hess J. (2019). Dengue fever in Saudi Arabia: A review of environmental and population factors impacting emergence and spread. Travel Med. Infect. Dis..

[24] Ashshi A.M. (2017). The prevalence of dengue virus serotypes in asymptomatic blood donors reveals the emergence of serotype 4 in Saudi Arabia. Virol. J..

[25] Abdullah M.A., Merdan A.I. (1995). Distribution and ecology of the mosquito fauna in the southwestern Saudi Arabia. J. Egypt. Soc. Parasitol..

[26] Al-Sheik A.A. (2011). Larval habitat, ecology, seasonal abundance and vectorial role in malaria transmission of Anopheles arabiensis in Jazan Region of Saudi Arabia. J. Egypt. Soc. Parasitol..

[27] Alahmed A.M., Al Kuriji M.A., Kheir S.M., Alahmedi S.A., Al Hatabbi M.J., Al Gashmari M.A. (2009). Mosquito fauna (Diptera: Culicidae) and seasonal activity in Makka Al Mukarramah Region, Saudi Arabia. J. Egypt. Soc. Parasitol..

[28] Aziz A.T., Dieng H., Ahmad A.H., Mahyoub J.A., Turkistani A.M., Mesed H., Koshike S., Satho T., Salmah M.C., Ahmad H. (2012). Household survey of container-breeding mosquitoes and climatic factors influencing the prevalence of Aedes aegypti (Diptera: Culicidae) in Makkah City, Saudi Arabia. Asian Pac. J. Trop. Biomed..

[29] World Health Organization (2019). Countries and Territories with Current or Previous Zika Virus Transmission.

[30] Aranda C., Sanchez-Seco M.P., Caceres F., Escosa R., Galvez J.C., Masia M., Marques E., Ruiz S., Alba A., Busquets N. (2009). Detection and monitoring of mosquito flaviviruses in Spain between 2001 and 2005. Vector-Borne Zoonotic Dis..

[31] Shimoda H., Hayasaka D., Yoshii K., Yokoyama M., Suzuki K., Kodera Y., Takeda T., Mizuno J., Noguchi K., Yonemitsu K. (2019). Detection of a novel tick-borne flavivirus and its serological surveillance. Ticks Tick borne Dis..

[32] Hobson-Peters J., Yam A.W., Lu J.W., Setoh Y.X., May F.J., Kurucz N., Walsh S., Prow N.A., Davis S.S., Weir R. (2013). A new insect-specific flavivirus from northern Australia suppresses replication of West Nile virus and Murray Valley encephalitis virus in co-infected mosquito cells. PLoS ONE.

[33] Guzman H., Contreras-Gutierrez M.A., Travassos da Rosa A.P.A., Nunes M.R.T., Cardoso J.F., Popov V.L., Young K.I., Savit C., Wood T.G., Widen S.G. (2018). Characterization of Three New Insect-Specific Flaviviruses: Their Relationship to the Mosquito-Borne Flavivirus Pathogens. Am. J. Trop. Med. Hyg..

[34] Memish Z.A., Zumla A., Alhakeem R.F., Assiri A., Turkestani A., Al Harby K.D., Alyemni M., Dhafar K., Gautret P., Barbeschi M. (2014). Hajj: Infectious disease surveillance and control. Lancet.

[35] Saudi Ministry of Health (2019). Health Requirements and Recommendations for Travellers to Saudi Arabia for Hajj and Umrah.

[36] Tambo E., El Dessouky A.G., Khater E.I.M. (2019). Innovative Preventive and Resilience Approaches Against Aedes-linked Vector-borne Arboviral Diseases Threat and Epidemics Burden in Gulf Council Countries. Oman Med. J..

